# The long-term outcome of patients with heroin use disorder/dual disorder (chronic psychosis) after admission to enhanced methadone maintenance

**DOI:** 10.1186/s12991-018-0185-3

**Published:** 2018-04-18

**Authors:** Angelo G. I. Maremmani, Alessandro Pallucchini, Luca Rovai, Silvia Bacciardi, Vincenza Spera, Marco Maiello, Giulio Perugi, Icro Maremmani

**Affiliations:** 1 Department of Psychiatry, North-Western Tuscany Local Health Unit, Versilian Zone, Viareggio, Italy; 2 AU-CNS, Association for the Application of Neuroscientific Knowledge to Social Aims, Pietrasanta, Lucca, Italy; 3 G. De Lisio Institute of Behavioural Sciences, Pisa, Italy; 4 School of Psychiatry, University of Pisa, Pisa, Italy; 5 Department of Psychiatry, North-Western Tuscany Local Health Unit, Apuan Zone, Massa, Italy; 6 Department of Clinical and Experimental Medicine, University of Pisa, Pisa, Italy; 7 Vincent P. Dole Dual Diagnosis Unit, Department of Specialty Medicine, Santa Chiara University Hospital, University of Pisa, Pisa, Italy

**Keywords:** Methadone maintenance, Long-term outcome, High-threshold methadone maintenance programme, Dual disorder, Chronic psychosis

## Abstract

**Background:**

Over-standard methadone doses are generally needed in the treatment of heroin use disorder (HUD) patients that display concomitant high-severity psychopathological symptomatology. A flexible dosing regimen may lead to higher retention rates in dual disorder (DD), as we demonstrated in bipolar 1 HUD patients, leading to outcomes that are as satisfactory as those of HUD patients without high-severity psychopathological symptomatology.

**Objective:**

This study aimed to compare the long-term outcomes of treatment-resistant chronic psychosis HUD patients (PSY-HUD) with those of peers without dual disorder (HUD).

**Methods:**

85 HUD patients who also met the criteria for treatment resistance—25 of them affected by chronic psychosis and 60 without DD—were monitored prospectively for up to 8 years while continuing to receive enhanced methadone maintenance treatment.

**Results:**

The rates of endurance in the treatment of PSY-HUD patients were 36%, compared with 34% for HUD patients (*p* = 0.872). After 3 years of treatment, these rates tended to become progressively more stable. PSY-HUD patients showed better outcome results than HUD patients regarding CGI severity (*p* < 0.001) and DSM-IV-GAF (*p* < 0.001). No differences were found regarding good toxicological outcomes or the methadone dosages used to achieve stabilization. The time required to stabilize PSY-HUD patients was shorter (*p* = 0.034).

**Conclusions:**

An enhanced methadone maintenance treatment seems to be equally effective in patients with PSY-HUD and those with HUD.

## Introduction

Opioid Use Disorder patients show a high rate of psychiatric comorbidities (anxiety, depression, sleep disorders) during agonist opioid treatment (AOT) [1]. The presence of psychotic and affective symptoms is a common feature of psychiatric disorders, and their link with substance use disorder (SUD) has been widely demonstrated in the literature [2]. Regarding patients with heroin use disorder (HUD), according to our previous studies, the onset of psychosis generally occurs before substance use begins, whereas affective symptoms develop afterwards [3, 4]. The natural history of HUD differs between psychotic and bipolar HUD patients, so these two categories of patients often present different clinical pictures at the moment of admission to their first Agonist Opioid Treatment (AOT). In HUD patients with chronic psychosis (PSY-HUD), the progression of the addictive disease is limited, whereas bipolar 1 HUD patients show a more severe substance (i.e. heroin) use illness [5]. In patients with PSY-HUD, a therapeutic use of heroin cannot be excluded, at least, at the beginning of their clinical history. This kind of situation was not reported in the case of bipolar HUD patients [4].

During AOT treatment, especially during methadone treatment, our patients with and without the diagnosis of a dual disorder (DD), considering DD patients as patients displaying SUD together with another concomitant mental illness, show extreme variability of the dose needed to prevent a relapse during treatment [6, 7]. In our patients, above-standard methadone doses are usually needed in the presence of high-severity psychopathological symptomatology characterized by somatization, depression, paranoid ideation and psychoticism [8]. Above-standard doses are likewise needed for DD patients [9]. Good outcomes are also related to methadone dosage in our previously studied bipolar 1 HUD patients [10].

The present study has aimed to compare the long-term outcomes of treatment-resistant PSY-HUD with those of HUD patients without psychiatric comorbidity (HUD). We decided to evaluate whether chronic psychosis was able to influence methadone treatment outcomes in patients who had previously been non-responders in front-line, low-threshold treatment facilities when those patients were included in a high-threshold, maintenance-oriented, high-dose methadone programme.

The study hypothesized that a diagnosis of chronic psychosis would not affect treatment outcomes if PSY-HUD patients received individualized (above-standard) doses of methadone, and that a good outcome would be related to long-term ongoing treatment (retention).

To test this hypothesis, PSY-HUD and HUD patients were followed in a naturalistic approach applied for up to 8 years in the context of the maintenance high-threshold, high-dose Pisa Methadone Programme, using retention in treatment and rates of heroin use as the primary end-point parameters.

## Methods

### Design of the study

A prospective cohort study was designed to evaluate the treatment outcome (in terms of retention in treatment, substance use, clinical improvement and general social adjustment) of patients included in a methadone programme, with reference to its relationship with chronic psychosis comorbidity.

### Setting

In Italy, low-threshold facilities for HUD patients are available in each territorial district. In those settings, when opioid agonists are employed, dosage and duration of treatment are usually limited, regardless of clinical indication [11], which suggests the value of increased dosage or treatment duration. Patients are allowed to negotiate the lowering of dosages, regardless of urinalyses, and to have their medication tapered earlier than advisable on the basis of the scientific literature.

All the patients participating in the study were treated in the setting of the Pisa Methadone Maintenance Treatment Programme (Pisa-MMTP) at Santa Chiara University Hospital, following the methodology proposed by Dole and Nyswander [12, 13].

Dole and Nyswander’s methodology involves four broad stages: (1) Induction—under medical supervision, the patient is transferred from street heroin to the maintenance medication. The induction phase involves an initial low dose, followed by titration over subsequent days to achieve a stable dose (which includes reaching a steady-state plasma concentration). (2) Stabilization—dose increments to deliver a maintenance dose that allows opioid withdrawal signs and symptoms to be alleviated without producing significant euphoria. (3) Maintenance—maintaining the patient on a stable regular dose of the medication. Monitoring is essential during this phase to monitor treatment progress and to change the dose level if necessary. Psychosocial interventions are offered during this period. (4) Medically supervised withdrawal—while retention in the treatment programme is an important target (with at least 12-month retention necessary for enduring positive changes to behaviour to be achieved), patients should be helped to withdraw from opioids if that is their informed choice. Safe withdrawal from the medication can be accomplished by gradual reductions in the dose—this minimizes the likelihood of significant withdrawal and allows time for neuronal re-adaptation to take place. After-care strategies, such as counselling and support, are developed at this time to maximize the possibility of enduring abstinence.

The length of each treatment phase, notably the stabilization and maintenance stages, can vary substantially among patients, with some patients remaining in the maintenance phase for years, or even a lifetime. Conversely, due to the chronic, relapsing nature of opioid dependence, many patients will not complete an entire treatment episode and may drop out at some point during the process. These patients may, after a while, begin a new treatment episode. Patients who have remained abstinent from drugs also may be liable to relapse into drug use. Thus, many opioid-dependent patients will enter treatment numerous times.

After patients at the PISA-MMTP had been safely inducted into treatment with methadone, their doses are gradually increased until the point is reached where there is no more than one urine drug screen which is positive for illicit opiates, cocaine or benzodiazepines in the previous 60-day period. Once this requirement is fulfilled, the patient is defined as having been “stabilized”, and the dose at which this goal has been accomplished is referred to as the ‘stabilization dose’. We consider these patients as reaching a good outcome. For more information, see Maremmani et al. [14].

### Participants

All PSY-HUD patients referred by low-threshold facilities for HUD patients to the Pisa-MMT programme during the January 1997–December 2006 period (*N* = 25) were consecutively enrolled in the study.

PSY-HUD patients were characterized by

- a diagnosis of HUD-concomitant chronic psychosis according to DSM-IV criteria for schizophrenia or delusional disorders,
- “absence of additional psychiatric DSM-IV diagnoses” and
- resistance to previous front-line, low-threshold methadone treatment programmes attended at local addiction treatment units.

Schizoaffective disorder, or bipolar disorder with psychotic features, was excluded from the PSY-HUD group because the present study aimed to discuss differences between PSY-HUD and HUD patients with reference to what we found in our previously studied bipolar 1 HUD patients.

We did not use a specific screening process other than the patient’s wish to be treated and wanting to participate. Criteria for treatment resistance included at least two unsuccessful treatments in the 2-year period before being referred to our programme. Patients had been treated with the standard protocols for HUD (methadone maintenance with dosages up to 100 mg/day) and were discharged because of persisting positivity for opioid metabolites at urinalyses.

Axis 2 diagnoses were excluded from the study, since a wide range of personality disorders was usually displayed by SUD patients, which makes it challenging to define Axis 2 diagnostic subgroups. Addictive behaviours may carry diagnostic implications, as in the case of borderline and antisocial personality disorders [15–19]. HUD patients co-affected by Borderline Personality Disorders and Antisocial Personality Disorder, during treatment or during detoxification programmes, maintain significantly higher levels of crime, injection-related health problems, heroin overdose, major depression and poorer global mental health [20–22].

As control group, we considered patients we had previously compared with our bipolar 1 HUD patients [10]. We excluded 3 patients because of a positive family history of psychiatric disorders and a diagnosis of affective temperament according to the criteria of Hagop Akiskal [23].

All patients gave their written informed consent to the study after the procedure had been fully explained. The consent form and the study protocol were both approved by the ethics committee of the University of Pisa, according to the WMA Declaration of Helsinki—Ethical Principles for Medical Research Involving Human Subjects.

### Instruments

#### Drug Addiction History Questionnaire (DAH-Q)

The DAH-Q [24] is a questionnaire comprising the following categories: sociodemographic information, physical health, mental health, substance use, treatment history, social adjustment and environmental factors. The questionnaire checks 10 areas: physical problems, mental problems, employment status, family situation, sexual problems, socialization and leisure time, legal problems, substance use, previous treatment and associated treatments. Items have been constructed in such a way as to ensure dichotomous (yes/no) answers. For more details, see [25]. The DAH-Q was administered at the beginning of treatment.

#### Global assessment of functioning, DSM-IV-GAF and clinical global impression (CGI)

The GAF considers psychological, social and occupational functioning within the sphere of a hypothetical mental health-illness continuum, without including any impairment of functioning due to physical or environmental limitations. The point allocation follows a specific code, with a maximum of 100 and a minimum of 0, with the possibility of using intermediate codes if necessary [26].

The CGI considers the severity of the disorder, the degree of improvement or worsening following the intervention and any adverse reactions [27].

CGI and GAF were administered monthly by a researcher who was blind as to the diagnosis of the patients.

#### Psychiatric diagnostic evaluation. Structured clinical interview for DSM-IV Axis I disorders (SCID-I)

This instrument [28] will help clinicians make standardized, reliable and accurate diagnoses while avoiding the common problem of premature closure (a premature focusing on one diagnostic possibility).

#### Toxicological urine analyses

The enzyme-multiplied immune technique for opiates was used. Toxicological urine analyses for morphine metabolites were carried out randomly every week during the induction phase and, randomly, almost every month, during the stabilization phase.

### Procedures

Patients were evaluated outside acute phases at the end of their first hospitalization at our clinic, so as to reduce the diagnostic ambiguity between intoxication-related symptoms and spontaneous (substance-unrelated) mental disorders. In cases where further information emerged on clinical grounds during the monitoring period, diagnoses were reviewed.

Patients who stayed in treatment were assessed at the end of treatment. Among patients with poor outcomes, those who left the treatment were assessed at the time of treatment interruption, this being the last regular assessment.

PSY-HUD patients were treated with low oral dosages of second-generation antipsychotics during the acute phase of a psychotic episode and until its resolution. Psychotropic medications were not systematically used for the HUD patients. In summary, no differences were detected either in anti-psychotic use or in psychosocial management in the PSY-HUD group when compared with their previous low-threshold treatment. Long-term treatment with antipsychotics was not our main choice, considering the negative impact of antipsychotics on the anti-reward syndrome of SUD patients [29, 30]. In any case, in our unit, top priority was given to methadone dosage adaptation [31].

In our programme, patients are required to attend the clinic according to scheduled appointments, to participate in the development of their treatment plan, to work towards treatment goals, to meet with medical and case management staff, and to attend groups whenever necessary.

Take-home doses, without limitations, at most for a 7-day period, are allowed, once patients have shown complete compliance with the rules of the programme. Every 7-day period, medication management visits were applied.

## Data analysis

Heroin use disorder patients and PSY-HUD patients were compared at treatment entry for demographic and addiction history using the Chi-square test for categorical variables, and Student’s *t* test (designed for Independent Samples) for continuous variables.

The association between differences in demographic data, addiction history and retention in treatment was tested using Cox regression. In our model, we included each difference at treatment entry between groups as an independent variable, and poor outcomes for individual patients as dependent variables.

Retention in treatment was analysed by means of the Kaplan–Meier survival analysis and Wilcoxon statistics for comparison between the survival curves. For the purpose of this analysis, the term ‘censored observations’ refers to patients who were still in treatment at the end of the study or were leaving treatment for reasons unrelated to the treatment itself (e.g. patients moving on to other therapeutic communities, due to imprisonment for old crimes). We considered it to be a poor outcome (terminal event) when a patient had not reached stabilization within a year or had relapsed into addictive behaviour after a period of stabilization, abandoned the treatment or been expelled from it.

The toxicological urinalyses were expressed using two indices: The TGO index (per cent Toxicological Good Outcome) and TGO ratio (per total specimens Toxicological Good Outcome). The TGO index expresses the percentage ratio between urinalyses proving negative for the presence of morphine and the total number of urinalyses carried out for each patient during the treatment period. It is the percentage ratio between the number of urinalyses testing negative for the presence of morphine and the number of urine analyses that the protocol has envisaged throughout the process. The TGO index tends to give preference to patients who remain ‘opiate-free’ but who terminate the study in advance for reasons not correlated with the study (for example, imprisonment). The TGO ratio further comprises how long the patient remains in the protocol, but gives less precedence to these patients. These two indices represent the two extremes, and the results tend to balance out. Concerning these parameters, the comparison between the two groups was made using ANOVA Two Factors (group and outcome).

Regarding global clinical impressions and social adjustment outcomes, we compared the two groups using ANOVA Two Factors (group and outcome) for cross-sectional evaluation and repeated analysis of variance for longitudinal evaluations.

Regarding stabilization methadone dose and the time required to reach the stabilization phase, we compared the two groups using ANOVA Two factors (group and outcome).

The statistical tests were considered significant at the level of *p* < 0.05.

## Results

### Baseline evaluation (at the beginning of the treatment)

Table 1 shows the demographic and clinical characteristics of participants. The discriminant characteristics of PSY-HUD patients were as follows. PSY-HUD patients more frequently had education lasting less than 8 years, presented a lower level of social adjustment with a lower frequency of legal problems, and self-reported a lower severity of drug addiction history. More specifically, PSY-HUD patients less frequently showed physical concerns and polysubstance use, talked about having experienced unsuccessful treatments or having received ongoing psychosocial treatments at local units, declared ‘daily or more’ heroin intake before requesting treatment and reached stage 3 of heroin addiction. Also, PSY-HUD patients were older when they first started using heroin and when they began their continuous use of heroin. Lastly, the duration of their dependence was shorter. Cox regression analysis using the variables reported above—showing differences between PSY-HUD and HUD patients—as predictors, and patients’ poor outcome as a criterion, did not show significant correlations except for a low educational level (HR = 3.72; 95% CI 1.69–8.16; *p* = 0.001), (*χ*^2^ = 22.54; d*f* = 13; *p* = 0.047).

**Table 1:** Demographic characteristics and drug addiction history of 85 heroin-dependent patients, comprising 25 with chronic psychosis (PSY-HUD) and 60 without Axis I psychiatric comorbidity (HUD)

	HUD patients *N* = 60	PSY-HUD patients *N* = 25	*t*/chi	*P*
Age (M ± s)	30.08 ± 5.9	30.64 ± 7.4	− 0.36	0.715
Gender: male [*N* (%)]	46 (76.2)	15 (60.0)	2.41	0.185
Marital status: single [*N* (%)]	47 (79.7)	16 (64.0)	2.29	0.169
Education: < 8 years [*N* (%)]	12 (21.1)	11 (44.0)	4.53	0.033
Work [*N* (%)]				
White collar	12 (20.7)	8 (32.0)		
Blue collar	18 (31.0)	10 (40.0)		
Unemployed	28 (48.3)	7 (28.0)	3.04	0.218
CGI severity of illness^a^ (*M* ± *s*)	5.54 ± 0.6	5.52 ± 0.7	0.12	0.900
GAF index from DSM-IV (*M* ± *s*)	44.75 ± 7.2	40.64 ± 10.6	2.06	0.042
1-Presence of physical concerns [*N* (%)]	48 (80.0)	14 (56.0)	5.15	0.023
2-Presence of altered mental status [*N* (%)]	54 (90.0)	25 (100.0)	2.69	0.101
3-Work concerns [*N* (%)]	42 (70.0)	14 (56.0)	1.53	0.215
4-Household concerns [*N* (%)]	14 (23.3)	9 (36.0)	1.43	0.231
5-Romantic concerns [*N* (%)]	24 (40.0)	13 (52.0)	1.03	0.309
6-Social/leisure concerns [*N* (%)]	33 (55.0)	12 (48.0)	0.34	0.556
7-Legal concerns [*N* (%)]	29 (48.3)	2 (8.0)	12.39	< 0.001
8-Polyabuse (more than 3) [*N* (%)]	45 (75.0)	11 (44.0)	7.454	0.006
9-Past treated [*N* (%)]	50 (83.3)	7 (28.0)	24.46	< 0.001
10-Combined treated [*N* (%)]	60 (100.0)	15 (60.0)	27.20	< 0.001
‘Daily or more’ heroin intake [*N* (%)]	55 (91.7)	14 (73.7)	4.22	0.040
Stable modality of use [*N* (%)]	21 (35.0)	12 (57.1)	3.15	0.076
Stage 3 of heroin addiction [*N* (%)]	47 (78.3)	5 (26.3)	17.37	< 0.001
Age at first use of heroin (*M* ± *s*)	18.22 ± 3.0	21.21 ± 5.0	− 3.16	0.002
HUD: age at onset (*M* ± *s*)	20.10 ± 3.9	24.75 ± 5.9	− 4.19	< 0.001
Dependence duration (years) (*M* ± *s*)	9.51 ± 6.1	4.15 ± 3.8	3.79	< 0.001
Age at first treatment (*M* ± *s*)	28.47 ± 6.8	30.64 ± 7.4	− 1.30	0.195

^a^Between 1 = normal and 7 = extremely ill

### Retention in treatment

Regarding the outcome of patients, as related to chronic psychotic comorbidity, at the end of the observational period, 12 (20.0%) HUD patients and 8 (32.0%) PSY-HUD patients completed their rehabilitation programme and either left the treatment or were referred to another programme as a “stabilized patient”. Thirty-two (53.3%) HUD patients and 15 (60.0%) PSY-HUD patients had not reached stabilization within a year or had relapsed into heroin use during the programme, so they were terminated and referred to their local treatment services. Sixteen (26.7%) HUD patients and 2 (8.0%) PSY-HUD patients were considered “stabilized” and were still in treatment at the end of the period of observation. These differences were not statistically significant (*χ*^2^ 4.12, d*f* = 2, *p* = 0.127). No patients left the treatment because of side effects. Four HUD patients (6.6%) and none of the PSY-HUD patients were dismissed for violence (*p* = 0.136); none were imprisoned or rehospitalized because of a psychotic episode.

**Fig. 1:**
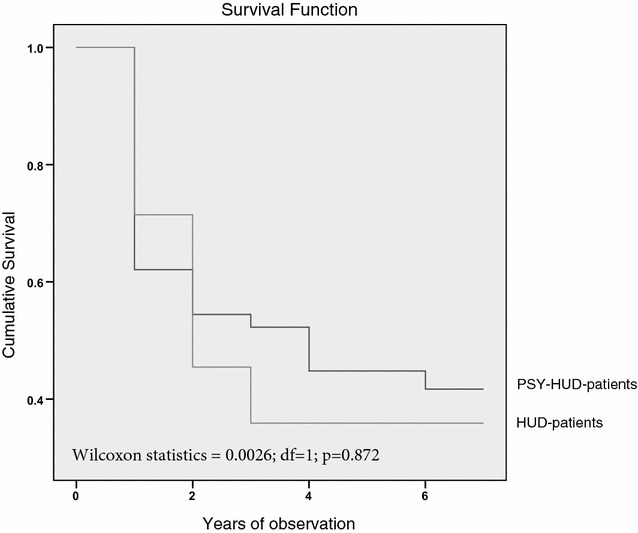
Endurance in methadone maintenance of 25 treatment-resistant heroin-dependent patients with chronic psychosis (PSY-HUD) and of 60 without DSM-IV Axis 1 psychiatric comorbidity (HUD)

Numbers of HUD patients entering annual intervals, according to the presence of chronic psychosis, are shown in Fig. 1. Twenty-two HUD patients (37%) and 7 PSY-HUD patients (28%) had not reached the stabilization phase in 1 year. None of the PSY-HUD patients relapsed into addictive behaviour (reusing heroin) after 3 years of treatment. In summary, according to the Kaplan–Meier methodology, the HUD patients’ Cumulative Proportion Retained (CPR) in treatment at the end of the observational period was 0.34. The proportion of PSY-HUD patients was 0.36. These differences were not statistically significant (*p* = 0.872).

Males (CPR = 0.36) and females (CPR = 0.30) showed a similar retention rate (Wilcoxon statistics = 0.50; d*f* = 1; *p* = 0.478). PSY-HUD (CPR = 0.33) and HUD (CPR = 0.37) males did not show a significantly different retention rate from HUD females (Wilcoxon statistics = 0.22; d*f* = 1; *p* = 0.635). The same results were observed in comparing PSY-HUD females (CPR = 0.40) and HUD ones (CPS = 0.27) (Wilcoxon statistics = 0.81; d*f* = 1; *p* = 0.367).

### Outcome

#### Urinalyses

When the toxicological examination performed at the time of enrolment into the programme (which was required to be positive) was eliminated from the analysis, 10,674 urine samples were analysed in all: 7963 for good-outcome patients, 2711 for poor-outcome patients. A total of 7885 samples from HUD patients were examined, with 2788 from PSY-HUD ones. On average, 74.54% of samples tested negative for morphine. No patient provided positive samples for the entire duration of treatment. No patient provided exclusively negative samples.

In good-outcome PSY-HUD patients, the TPO index was 91.60 ± 4.2; in good-outcome HUD patients, it was 87.43 ± 8.5 (Group effect: *F* = 1.13; d*f* = 1; p = 0.290; outcome effect: *F* = 146.78, *p* < 0.001; group-outcome effect: *F* = 0.74, *p* = 0.391). In good-outcome PSY-HUD patients, the TPO ratio was 0.47 ± 0.2; in good-outcome HUD ones, it was 0.49 ± 0.3 (Group effect: *F* = 0.09; d*f* = 1; *p* = 0.758; outcome effect: *F* = 46.32, *p* < 0.001; group-outcome effect: *F* = 0.00, *p* = 0.978). In summary, no differences were found regarding urinalyses for morphine between PSY-HUD and HUD patients during the observational period.

#### Global clinical impressions and social adjustment outcomes

The CGI severity of illness and the DSM-IV GAF (global assessment of functioning) showed the following significant trends in participants.

At the end-point evaluation, good-outcome PSY-HUD patients reported a lower severity of illness (1.90 ± 0.9) than good-outcome HUD patients (2.46 ± 0.7). Time effect (*F* = 3303.54; d*f* = 1; *p* < 0.001) and group-time effect (*F* = 48.38; d*f* = 1; *p* < 0.001) were significant. Interestingly, at baseline, the severity of illness was equal in the two groups. These differences were not related to the outcome (*F* = 0.55; d*f* = 1; *p* = 0.458).

At the end-point evaluation, good-outcome PSY-HUD patients reported a better level of social adjustment (78.30 ± 8.1) than good-outcome HUD patients (74.39 ± 9.8). Time effect (*F* = 376.01; d*f* = 1; *p* < 0.001) and group-time effect (*F* = 8.90; d*f* = 1; *p* = 0.004) were both significant. These differences were not related to the outcome (*F* = 0.05; d*f* = 1; *p* = 0.824).

#### Stabilization methadone dose and time to reach the stabilization phase

On average, PSY-HUD (115.20 ± 41.2 mg/day; 60–190 ranged) and HUD patients (120.18 ± 67.4 mg/day; 30–260 ranged) did not need statistically different methadone dosages in the stabilization phase (*F* = 0.28; *p* = 0.597). Differences between groups were not observed even when dosage was controlled by the outcome (*F* = 0.45; *p* = 0.502).

On average, PSY-HUD patients needed 2.76 ± 0.9 months to reach the stabilization dosages; otherwise, HUD patients were stabilized in 6.35 ± 9.3 months (Group effect: *F* = 4.65, *p* = 0.034; Outcome effect: *F* = 2.60, *p* = 0.111; Group-outcome effect: *F* = 4.15, *p* = 0.045).

## Discussion

We have examined treatment retention and outcomes for PSY-HUD and HUD patients involved in an enhanced methadone treatment. We noted that:

- At baseline, PSY-HUD patients more frequently showed education lasting less than 8 years, a lower level of social adjustment and legal problems, and a lower severity of drug addiction history. These characteristics did not appear to be related to better retention or better outcome, the only exception being the low educational level.
- PSY-HUD and HUD patients were retained in treatment without differences. Males and females showed similar retention rates.
- Between groups, no differences were found regarding urinalyses for morphine or regarding methadone stabilization dosages.
- The time required to stabilize PSY-HUD patients was shorter.

It is not easy to correlate low educational level with poor outcome. It is known that, in SUD patients, successful treatment was associated with several baseline characteristics including older age, white race, having more than a high school education, lower level of care and not having a history of opioid use [32]. It is also true that, at first sight, control patients seem to be a more seriously ill control group. We assume, however, that only the drug-related history is less severe in PSY-HUD patients. The overall clinical judgment expressed by CGI stresses the same severity of disease in both groups. In addition, there is no doubt that from the psychopathological point of view, PSY-HUD patients are more seriously ill than their peers without DD.

In our study, the outcome and the retention rate in the therapy of treatment-resistant participants did not differ from that of long-term standard methadone programmes designed according to the methodology of Dole and Nyswander [33–35]. Participants were stabilized using middle-to-high dosages, but not with the above-standard dosages we used to stabilize bipolar 1 HUD patients [10].

Anecdotal evidence has been reported about the beneficial effects of opiates in reducing psychotic symptoms. In a 39-year-old man requesting treatment for positive psychotic symptoms, a low dose of quetiapine and 140 mg daily of methadone had controlled psychosis for years. After interrupting the use of anti-psychotic medication and methadone for complaints in the sexual area, he presented acute psychotic symptoms. After he started taking heroin regularly, treatment with methadone and quetiapine was resumed, and his symptoms subsided. A few months later, he again stopped using methadone, without relapsing into heroin use; his psychotic symptoms reappeared, even though he maintained the anti-psychotic medication [36]. The use of high methadone dosage has been confirmed in a series of psychotic HUD patients [37] and one patient responded to an increased dose of methadone [38]. Reports of the occurrence of a psychotic episode after methadone or buprenorphine discontinuation are, in the literature, a little bit more frequent [39–41].

Higher methadone doses have been used in Comorbid Psychiatric Disorder [42], as an anti-anxiety, anti-depressant and anti-psychotic treatment [43].

In our study, a possible explanation for the need for these relatively higher doses in patients who had previously been unresponsive to standard treatments may be related to a wide inter-individual variability in the methadone metabolism [44, 45], which may explain why a number of patients are under-medicated, if a standard (middle-to-low) dose of methadone is used. Unfortunately, we did not measure plasma methadone levels in studying participants during the stabilization phase, so we cannot resolve this doubt.

In study participants, only Axis I psychiatric disorders were taken into consideration, and the existence of a minor form of psychopathology in the other patients concealed under the main addictive symptoms cannot be excluded. We refer to the psychopathological symptoms of Axis II psychiatric disorders and/or psychopathological symptoms related to the HUD [46]. Participants were, however, followed up for a long time (up to 8 years), and diagnoses were subject to revision whenever further clinical evidence or retrospective information was gathered—a factor that reduces the likelihood of false HUD. Moreover, the duration of addiction was such as to make it improbable that participants rated as HUD had a silent psychiatric history. The availability of significant others was itself extremely helpful in increasing the level of diagnostic accuracy and grouping.

The high GAF score values recorded for PSY-HUD participants without hospitalizations throughout the treatment period showed that participants were simultaneously compliant both with MMT requirements and with the specific therapy adopted for their comorbid psychopathology. The use of new-generation antipsychotics for the treatment of psychiatric symptoms—medication not wholly changed by the need to treat addiction—may partly explain the positive outcomes obtained in psychotic participants. This effect cannot be attributed exclusively to the effects of pharmacotherapy. A lack whether of appropriately flexible methadone doses and/or of specific medications given in association with methadone treatment for PSY-HUD patients could have been responsible for the conflicting results obtained by other researchers, who reported that psychiatric disorders were linked to worse treatment outcomes (such as drug use and criminal activities) [47–49]. Also, the high therapeutic pressure associated with our programme could have been responsible for better results [50].

In the present study, methadone is potentially useful in treating psychosis, at least in HUD patients. This is in line with a series of observations about the correlation between opioid use and psychosis [51].

We are aware that it is very difficult to find published papers about the dosages of AO medications in dual-disorder patients, to corroborate the results of the present study. Our present results do, however, look stronger in the light of our previous studies, with all the limitations that this fact entails. The concern is that we may be overinterpreting our previous research despite the fact that it consisted of single site, small sample size, homogeneous population studies carried out in Europe (selected for patients who had failed to achieve results at lower levels of treatment). In the literature, there are still insufficient data to generalize the present results to cover all HUD patients with comorbid non-affective psychotic disorders. We wonder, of course, if our findings would apply to other HUD sub-populations (e.g. adolescents or psychiatric patients with HUD not seeking substance use disorder treatment). In any case, our previous studies do allow us to present some afterthoughts on the results of our present study.

In HUD patients admitted to hospital for an acute psychotic episode, we found that an increase in methadone dosage or the initiation of methadone treatment proved to be potentially effective in achieving control of psychotic symptoms by prescribing lower treatment dosages of antipsychotics and mood-stabilizing drugs, even when the period spent in hospital was the same [52].

We also found that the profile of psychotic HUD patients at their first treatment attempt displays a higher level of global symptom severity, even when coupled with less severe addictive symptoms and a shorter duration of addictive history than their non-psychotic peers [53].

Our psychotic HUD patients may be included among those who resort to street methadone as a regular practice before entering treatment, and this decision should be regarded as a self-harm-reducing behaviour rather than a pattern of use. Our patients may, in fact, have an independent motivation to look for treatment earlier and stay in treatment longer—an advantage that may overcome addictive ambivalence and improve compliance [54].

We also found that there is a distinction to be made between patients who had started heroin use after the onset of psychiatric disorders and those who had suffered from psychiatric disorders after the onset of their drug habit. Among the former, psychotic disorders and anxiety disorders were those best represented, and they were linked with a trend towards less severe addictive symptoms. The latter group mostly comprised patients with mood disorders, who had more severe addictive symptoms. This time sequence does not stand as a definite proof of self-medication dynamics, but it is broadly consistent with the idea that some disorders, rather than others, may lead to heroin use in a self-medication manner [3]. The same patients would then suffer from early impairment of their psychiatric disorders, due to acquired opiate imbalance, when the severity of their addictive disease is still lower, and they will benefit more directly from the opiate-balancing effect of agonist treatment [55].

Through the recent use of an exploratory factor analysis of the 90 items in the SCL-90, a five-factor solution was identified for HUD patients, namely “Worthlessness and Being Trapped”, “Somatic Symptoms”, “Sensitivity-Psychoticism”, “Panic Anxiety” and “Violence-Suicide” [46]. Using this SCL-90, 5-factor solution, our HUD patients with prominently psychopathological “Sensitivity-Psychoticism” characteristics showed a better level of retention in treatment when treated with methadone [56].

According to our research group, methadone dosage would partly work as a psychotropic stabilizer, regardless of addictive symptoms, so that the eventual stabilization dosage is higher than in non-psychotic HUD patients. Once both psychopathological grounds (addictive and psychotic) have been neutralized, many HUD patients may achieve a positive outcome, reversing what might be expected in the absence of treatment [57, 58].

Lastly, in the present study, methadone should accelerate the stabilization process in psychotic HUD patients through the early normalizing of the opioid system.

Generally, patients requiring high-dose methadone are polydrug SUD patients or patients with psychiatric comorbidities [59]. That result was confirmed by us in HUD bipolar 1 patients [10], but not in psychotic ones, in which standard doses seem to be potentially effective, too. We should, in fact, keep in mind that an overall improvement in the psychiatric status of HUD patients has been reported, independently of the dosages used [60], and that a very high prevalence of psychiatric comorbidity is present in HUD patients receiving OAT [1].

### Limitations

In any case, the validity of our study was limited by several factors—primarily, the observational nature of the protocol. Observational studies do, however, have the merit of capturing the most clinically significant data, such as data on the effectiveness and toxicity of therapies used by a heterogeneous population of patients in the ‘real world’.

During the maintenance phase of the treatment, urine screening for cocaine and benzodiazepine was not performed systematically but randomly, every 6 months. All patients were evaluated almost at the same time, and patients with the presence of urine metabolites for cocaine and benzodiazepine were considered to be poor responders. Cannabinoid use was not assessed. The strength of the study was, however, the absence, in our patients, of cocaine or benzodiazepine comorbid diagnosis according to the DSM-IV-R criteria and the absence of cocaine and benzodiazepine use in all stabilized patients.

The sample size of this study was insufficient in number, and at the end of the observational period, only two participants were present in the DD group. In addition, it was not possible to have a follow-up evaluation in the case of the participants who dropped out. Of course, this small population and the fact that the majority of the patients have left the study before the end makes statistical analysis fairly tricky. This also makes it difficult to formulate any associations between population characteristics and treatment retention.

Lastly, one cannot fail to consider the multiple interferences caused by inter-individual variability (presence of mental disorder and personality traits and their neurobiological correlates), the possible selection biases, the clinical setting and the temporary use of adjunctive medications.

## Conclusions

Opioid agonists deserve reconsideration, not only because of their anti-craving capability but also because of their helpfulness at the psychopathological level. They represent an adequate tool, even in the task of treating psychiatric symptomatology and psychiatric disorders, especially chronic psychosis, in HUD patients. In this case, a flexible dosing regimen that permits the administration of standard (middle-to-high) methadone doses may lead to satisfactory outcomes and to a retention rate very similar to that of HUD patients without other mental disorders while restraining psychotic symptoms and reducing the risk of rehospitalization. In conclusion, personalized methadone treatment seems to be capable of producing a good result in treatment-resistant HUD patients with or without chronic psychosis.
